# Associations between self-reported SARS-CoV-2 infection status, serology and common longer-term COVID-19 symptoms among adults in Canada, a cross-sectional study

**DOI:** 10.14745/ccdr.v51i04a05

**Published:** 2025-04-03

**Authors:** Alain Demers, Dianne Zakaria, Nicholas Cheta, Peri Abdullah, Samina Aziz

**Affiliations:** 1 Health Promotion and Chronic Disease Prevention Branch, Centre for Surveillance and Applied Research, Public Health Agency of Canada, Ottawa, ON; 2 Department of Community Health Sciences, University of Manitoba, Winnipeg, MB

**Keywords:** SARS-CoV-2, COVID-19, post-COVID condition, antibodies, cross-sectional survey

## Abstract

**Background:**

A variety of methods, including self-report and antibody testing, has been used to estimate the prevalence of SARS-CoV-2 infections and related longer-term symptoms, but the impact of employed methods on conclusions has not been thoroughly explored.

**Objective:**

We examined associations between self-report and antibody findings in the Canadian adult (aged 18 years and older) population.

**Methods:**

We used data from a large population-based cross-sectional probability survey conducted between April and August 2022. Self-reported infection status and experiences with common longer-term COVID-19 symptoms since the start of the pandemic was collected, as well as a dried blood spot to measure SARS-CoV-2 antibodies.

**Results:**

As of August 2022, the number of adults reported having had a confirmed or suspected infection was 37.9% (95% CI: 36.8%–39.1%), while the overall mean probability of having infection-related antibodies was 52.9% (95% CI: 51.8%–54.0%) and increased with respondent certainty they had been infected. However, the mean probability of having infection-related antibodies was not associated with infection severity or the reporting of common longer-term COVID-19 symptoms. More than one in five adults were unaware they had been infected.

**Conclusion:**

Self-report surveys may misclassify the SARS-CoV-2 infection status of a substantial proportion of untested people and may bias estimates of the percentage infected, the severity of infections and the risk of developing infection-related longer-term symptoms. Common longer-term COVID-19 symptoms reported by some could have been caused by other infections or diseases.

## Introduction

An estimated 16.0% of Canadians who self-report a confirmed or suspected SARS-CoV-2 infection develop post COVID-19 condition (PCC), also known as long COVID ((1)). Even people with mild or asymptomatic infections are at risk of developing PCC, although to a lesser extent than those with more severe symptoms ((2–4)). However, these longer-term symptoms attributed to COVID-19 can be generated by other infections or diseases ((5)). Consequently, in the absence of a control group, the occurrence of PCC could be overestimated in a population ((6)). A variety of methods, including self-report and antibody testing, have been used to estimate the prevalence of SARS-CoV-2 infections and related longer-term symptoms, but the impact of employed methods on conclusions has not been thoroughly explored ((7)). The second cycle of the Canadian COVID-19 Antibody and Health Survey (CCAHS-2) provides an opportunity to address this knowledge gap. In addition to self-reported infection status, the survey captured information on SARS-CoV-2 antibodies due to infection and asked all respondents, irrespective of infection status, about their experiences with common longer-term COVID-19 symptoms.

The objectives of this study are to describe the self-reported SARS-CoV-2 infection status and antibody test results of adults who were surveyed between April and August 2022, quantify the agreement between self-reported SARS-CoV-2 infection status and the presence of antibodies indicating a past infection and examine how self-reported common longer-term COVID-19 symptoms vary by self-reported infection status and antibody results. Our findings contribute to an evidence base that can be used to support the interpretation of research that relies on serology or self-report when assessing the burden of SARS-CoV-2 infections and PCC.

## Methods

### Data source

The CCAHS-2 is a large population-based cross-sectional multistage probability survey of the Canadian adult population, aged 18 years and older, living in private dwellings across the 10 provinces that was conducted between April and August 2022 ((8)). In addition to using an electronic questionnaire to capture information on SARS-CoV-2 infection history and common longer-term symptoms of COVID-19 since the start of the pandemic, the survey collected a dried blood spot (DBS) sample to measure the presence of antibodies against SARS-CoV-2 from vaccination or prior infection. Over 100,000 (n=105,998) adults were invited to participate and 15,701 completed at least part of the electronic questionnaire, provided a DBS sample and agreed to share their data with the Public Health Agency of Canada for an overall response rate of 14.8%. Except for antibody testing, results presented are based on self-reports and relate to the first SARS-CoV-2 infection with a positive test result or, in the absence of a positive test result, the first suspected infection. Additional methodological considerations and relevant questions that were asked to respondents are available in the **Appendix**.

### Probability of the presence of antibodies from a past SARS-CoV-2 infection

Statistics Canada derived a variable that specifically estimated the probability of the presence of antibodies from a past SARS-CoV-2 infection. This was done using information on the presence and quantity of nucleocapsid, spike and receptor binding domain of spike antibodies combined with results from a nucleocapsid cross-calibration study involving the two laboratories that conducted all the DBS sample testing. Briefly, spike and receptor binding domain of spike antibody positivity were based on lab-specific predetermined thresholds. If both tests were negative, the probability of the presence of antibodies from a past infection was zero. If either of these two tests were positive and the nucleocapsid titre value was below the lower limit of detection or lab-specific lower threshold, or above the upper limit of detection or lab-specific upper threshold, the probability of the presence of antibodies from a past infection was set to zero and one, respectively. For all other nucleocapsid titre values, a generalized linear model, developed using the cross-calibration study results, was used to estimate the probability of the presence of antibodies from a past infection. The cross-calibration study was conducted to address a misalignment between the two labs when assessing nucleocapsid titre values and thus standardize results ((8)). Since the accuracy of antibody tests are dependent on the time between infection and testing, a self-reported positive polymerase chain reaction (PCR) or rapid antigen test (RAT) was considered a more valid indicator of past infection when compared with the DBS result ((9)).

### Defining self-reported infection status and common longer-term symptoms of COVID-19

Based on responses to questions about ever testing positive for COVID-19 or suspecting an infection, respondents were classified into one of four mutually exclusive self-reported infection categories: confirmed infection, suspected infection, no suspected infection and uncertain infection. Adults responding that they did not know if they had been infected formed the “uncertain infection” group (see questions CS_Q05 and CS_Q15 in the Appendix). Respondents self-reporting a confirmed or suspected infection were asked about the severity of their acute infection symptoms (see question CS_Q40 in the Appendix) and whether they had experienced any symptoms three or more months after being infected, while all others were asked about any new unexplained symptoms lasting two or more months since the start of the pandemic. Those reporting symptoms were asked about their experiences with 13 common longer-term COVID-19 symptoms (see question CS_Q55 in Appendix), as well as the duration of these symptoms. Those reporting symptoms lasting three or more months were considered to have experienced common longer-term COVID-19 symptoms. We used a symptom duration of three months rather than symptoms present three or more months after infection to allow valid comparisons between adults with different self-reported infection statuses. Participants not completing the longer-term symptom sections or stating they did not know if they had experienced longer-term symptoms were assumed to have missing information regarding longer-term symptoms; these individuals accounted for 4.3% of the unweighted sample.

## Analyses

Analyses were conducted using SAS 9.4^TM^ Software with a two-tailed alpha level of 0.05. SAS survey procedures and bootstrap weights provided by Statistics Canada were used to produce estimates, 95% confidence intervals (CI) and tests of association that acknowledge the complex survey design through the bootstrap method. We used weighted means to estimate the probability of antibodies from a SARS-CoV-2 infection by self-reported infection status, days since first self-reported positive COVID-19 test, development of longer-term symptoms and severity of acute infection symptoms. Confidence intervals for weighted means use the t-distribution and tests for differences between group means use the t-test adjusted for multiple comparisons using the Bonferroni method. We used weighted proportions to estimate the percentage of adults reporting common longer-term COVID-19 symptoms by self-reported infection status and severity of acute infection symptoms. Confidence intervals for weighted proportions were calculated using the Clopper-Pearson (exact) method and the design-based first-order Rao-Scott test of association was used to test for group differences. Respondents with missing information were excluded from the analyses.

Our study was exempt from research ethics board review under article 2.2 of the *Tri-Council Policy Statement: Ethical Conduct for Research Involving Humans* ((10)).

## Results

**Figure 1:**
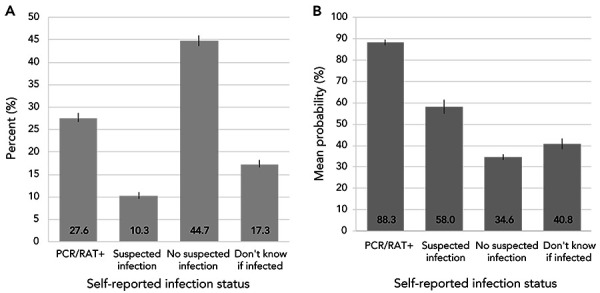
A) Distribution of self-reported SARS-CoV-2 infection status and B) mean probability of having antibodies consistent with a SARS-CoV-2 infection by self-reported infection status, April–August 2022^a,b,c^ Abbreviations: PCR, polymerase chain reaction; RAT, rapid antigen test ^a^ PCR/RAT+: self-reported a SARS-CoV-2 infection confirmed by PCR or RAT; suspected infection: self-reported a SARS-CoV-2 infection not confirmed by PCR or RAT; no suspected infection: did not self-report a SARS-CoV-2 infection ^b^ Source: Canadian COVID-19 Antibody and Health Survey Cycle 2 ^c^ Estimates for Canada exclude the Territories. All estimates are weighted percentages. The percentage of adults in Canada who were unaware they had been infected was estimated as follows: 0.346*0.447+0.408*0.173=0.225

**Figure 2:**
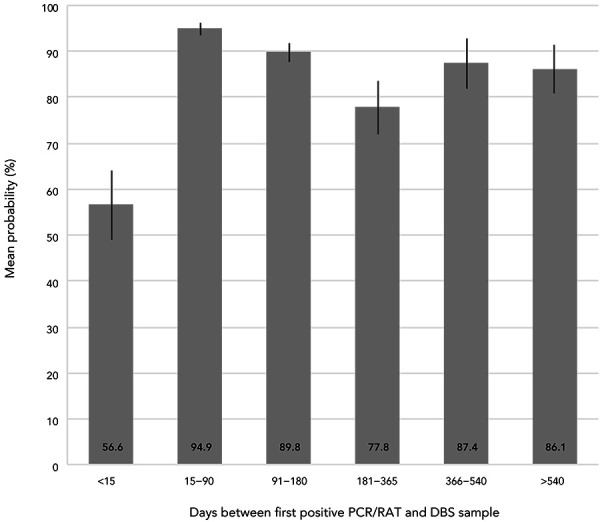
Mean probability of having antibodies consistent with a SARS-CoV-2 infection by days since first self-reported positive polymerase chain reaction/rapid antigen test, April–August 2022^a,b^ Abbreviations: DBS, dried blood spot; PCR, polymerase chain reaction; RAT, rapid antigen test ^a^ Source: Canadian COVID-19 Antibody and Health Survey Cycle 2 ^b^ Estimates for Canada exclude the territories. All estimates are weighted percentages

As of August 2022, 37.9% (95% CI: 36.8%–39.1%) of adults in Canada reported having had a confirmed or suspected infection (**Figure 1**, A), while the overall mean probability of having infection-related antibodies was 52.9% (95% CI: 51.8%–54.0%) and increased with respondent certainty they had been infected (Figure 1, B)). However, the mean probability of not having infection-related antibodies was 11.7% (95% CI: 10.5%–13.0%) for adults self-reporting a positive COVID-19 test, suggesting that DBS testing may miss infections and underestimate the prevalence of past infections in the general population. This is supported by the observation that the mean probability of having infection-related antibodies was lowest within two weeks of a positive COVID-19 test, highest 15 to 90 days after the test and slightly declined thereafter (**Figure 2**). Even though DBS test results underestimated the percentage of adults with past SARS-CoV-2 infections, the data still indicated that, on average, more than one in five adults in Canada were unaware they had been infected (Figure 1).

**Figure 3:**
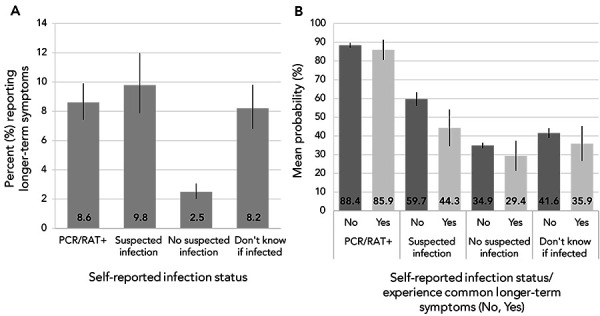
A) Percentage experiencing common longer-term COVID-19 symptoms, B) Mean probability of having antibodies consistent with a SARS-CoV-2 infection by self-reported infection status and experiences with common longer-term COVID-19 symptoms, April–August 2022^a,b,c^ Abbreviations: PCR, polymerase chain reaction; RAT, rapid antigen test ^a^ PCR/RAT+: self-reported a SARS-CoV-2 infection confirmed by PCR or RAT; suspected infection: self-reported a SARS-CoV-2 infection not confirmed by PCR or RAT; no suspected infection: did not self-report a SARS-CoV-2 infection ^b^ Source: Canadian COVID-19 Antibody and Health Survey Cycle 2 ^c^ Estimates for Canada exclude the territories. All estimates are weighted percentages

As of August 2022, 8.6%–9.8% of adults with a confirmed, suspected or uncertain infection status had ever experienced longer-term COVID-19 symptoms, more than three times that reported by adults with no suspected infection (2.5%) (**Figure 3**, A). Across self-reported infection statuses, however, the mean probability of having infection-related antibodies did not significantly differ by self-reported longer-term COVID-19 symptoms, except for those with suspected infections where adults with symptoms had a lower mean probability of having infection-related antibodies (44.3% vs. 59.7%, *p*=0.0036, Figure 2, B).

**Figure 4:**
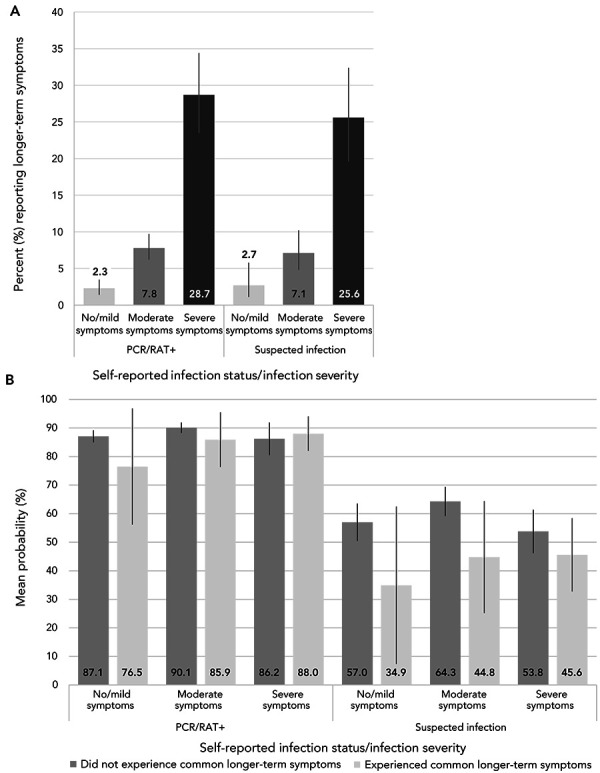
A) Percentage reporting common longer-term COVID-19 symptoms; B) Mean probability of having antibodies consistent with a SARS-CoV-2 infection, April–August 2022^a,b,c,d^ Abbreviations: PCR, polymerase chain reaction; RAT, rapid antigen test ^a^ PCR/RAT+: self-reported a SARS-CoV-2 infection confirmed by PCR or RAT; suspected infection: self-reported a SARS-CoV-2 infection not confirmed by PCR or RAT ^b^ Severity of initial infection was defined as follows: no or mild symptoms, didn’t affect my daily life; moderate symptoms—some effect on my daily life; severe symptoms, significant effect on my daily life or hospitalized due to symptoms ^c^ Source: Canadian COVID-19 Antibody and Health Survey Cycle 2 ^d^ Estimates for Canada exclude the territories. All estimates are weighted percentages

For both confirmed and suspected infections, the percentage of adults experiencing longer-term symptoms increased significantly with the severity of the initial infection symptoms (**Figure 4**, A). However, the severity of infection did not significantly influence the mean probability of having infection-related antibodies, irrespective of symptom presence (Figure 4, B). It is important to note the uncertainty around some of these results conveyed by the wide confidence intervals; thus, these estimates should be interpreted cautiously.

## Discussion

In a population-based probability sample of adults in Canada, we found that the mean probability of having infection-related antibodies increased with respondent certainty they had been infected but varied with the time interval between infection and DBS sample ((9,11)). We also found that more than one in five adults were unaware they had antibodies consistent with a SARS-CoV-2 infection and that the mean probability of having infection-related antibodies was not associated with the severity of infection or the reporting of common longer-term COVID-19 symptoms.

Our findings are consistent with other research examining the relationship between time since infection and antibody test results ((9,11)) and a recent systematic review that found no consistent relationship between the presence of longer-term symptoms and antibodies from a past SARS-CoV-2 infection irrespective of severity of acute infection ((12)). The lack of association between symptoms and infection-related antibodies suggests that the common longer-term COVID-19 symptoms reported by some adults could have been caused by other infections or diseases and is consistent with findings from a prospective population-based observational study that compared the prevalence and severity of symptoms before and after SARS-CoV-2 infection as well as with matched uninfected controls ((13)). Future work using CCAHS-2 data will explore these relationships using multivariable methods that acknowledge important covariates such as sex, age and pre-existing chronic conditions.

Our findings have implications for studies based solely on people who report a previous positive COVID-19 test or suspected infection. First, estimates of the percentage of people infected will be biased downward: the percentage of adults in Canada reporting a SARS-CoV-2 infection based on self-reported confirmed or suspected infections was about 37.9% (95% CI: 36.8%–39.1%) vs. a conservative estimate of 52.9% (95% CI: 51.8%–54.0%) based on DBS testing. Second, descriptions of the severity of acute SARS-CoV-2 infections may be biased upward as they exclude adults not suspecting an infection or uncertain of their infection status who were infected. For these groups, the mean probability of having infection-related antibodies was 34.6% and 40.8%, respectively (Figure 1, B). Last, estimates of the percentage of people developing longer-term symptoms after infection may be biased as a substantial proportion of adults with suspected infections do not have infection-related antibodies and those unaware of their infections will be excluded. Our results also indicate that studies based solely on serology will also underestimate the percentage of adults infected, particularly in the early post-infection period and as time since infection increases resulting in antibody waning.

## Strengths and limitations

The primary strengths of this study are that it 1) is population-based and 2) considers both self-reported information and measured SARS-CoV-2 antibody levels. However, several limitations should be considered when interpreting our findings. The CCAHS-2 collection and reference periods spanned a long time period during which the COVID-19 landscape across Canada changed substantially. Consequently, caution is warranted when comparing results to other studies with different time frames. The CCAHS-2 did not account for multiple infections in the same person, so common longer-term COVID-19 symptoms and antibody levels may not relate to the first self-reported SARS-CoV-2 infection. Estimates of the prevalence of common longer-term COVID-19 symptoms may be biased downward as respondents reporting more recent infections or occurrence of new unexplained symptoms would not have had adequate time to satisfy the three-month duration criterion. The accuracy of testing for antibodies can be impacted by a variety of factors including the timing of sample collection relative to infection and the number of antigens tested for. Testing too early or too long after infection may fail to detect infections due to the time needed to develop an immune response and antibody waning, respectively ((9,11)). Testing for three antigens, as was done in CCAHS-2, increases the accuracy of testing ((14)). Finally, a limited list of PCC symptoms was used and the inclusion of “other” symptoms as common longer-term symptoms of COVID-19 might be considered questionable. Nonetheless, 89.3% (95% CI: 85.9%–92.2%) of adults reporting longer-term symptoms experienced at least one of the specific symptoms.

## Conclusion

Self-report surveys may misclassify the SARS-CoV-2 infection status of a substantial proportion of untested people and may bias estimates of the percentage infected, the severity of infections and the risk of developing infection-related longer-term symptoms. The lack of association between common longer-term COVID-19 symptoms and the mean probability of having infection-related antibodies suggests that, for some, these symptoms could have been caused by other infections or diseases.

## Appendix

Methodological considerations and extracts from the questionnaire

**Methodological consideration: Respondents were asked about their SARS-CoV-2 infection history and common longer-term symptoms of COVID-19 since the beginning of the pandemic. However, the electronic questionnaire and dried blood spot sample were completed between April and August 2022.** Questions extracted from the electronic questionnaire available from: https://www23.statcan.gc.ca/imdb/p3Instr.pl?Function=assembleInstr&lang=en&Item_Id=1389871

There are two common types of COVID-19 tests. The first is a polymerase chain reaction (PCR) test, often used in health care settings, that is sent to a lab and produces the most accurate result. The second type is a rapid antigen test, often called a rapid test, which produces a result within minutes.

**CS_Q05.** Have you ever had a positive result on a COVID-19 test?

1. Yes

**CS_Q08.** Have you ever had a positive result on a PCR (lab) type COVID-19 test?

1. Yes

2. No

3. Don’t know

**CS_Q10.** Were you hospitalized for COVID-19?

1. Yes

2. No

2. No

3. Waiting for results

**CS_Q15.** Do you think you have ever had COVID-19?

Methodological consideration: Respondents that answered “don’t know” to CS_Q15 were put in the “uncertain infection” category.

1. Yes

2. No

3. Don’t know

**CS_Q40**. [When you had your first positive test/When you thought you first had COVID-19], how severe were your symptoms?

Methodological consideration: As indicated in the note to Figure 4, respondents hospitalized for COVID-19 (see CS_Q10) were included in the severe symptoms category.

1. No symptoms

2. Mild symptoms – didn’t affect my daily life

3. Moderate symptoms – some effect on my daily life

4. Severe symptoms – significant effect on my daily life

**CS_Q45**. Did you experience any symptoms 3 or more months after [your first COVID-19 symptoms started/your first positive test/you thought you first had COVID-19]?

Methodological consideration: Respondents selecting “yes” were classified as having longer-term symptoms if they subsequently reported a symptom duration of 3 or more months.

1. Yes

2. No

3. I [started feeling symptoms/had a first positive test/think I first had COVID-19] less than 3 months ago

**CS_Q55.** What symptoms did you experience 3 or more months after [your first COVID-19 symptoms started/your first positive test/you thought you first had COVID-19]?

Methodological consideration: Respondents that selected at least one of the following symptoms we considered has having reported longer-term symptoms.

Include symptoms from your initial infection that lasted 3 or more months or symptoms that developed after an initial recovery.

Select all that apply.

1. Fatigue, tiredness or loss of energy

2. Difficulty thinking or problem solving (brain fog)

3. Shortness of breath or difficulty breathing

4. Coughing

5. Fever

6. Chest pain

7. Stress or anxiety

8. Sadness, pessimism, hopelessness or depression

9. Pain (e.g., muscular, abdominal, joint; exclude chest pain or headache)

10. Symptoms relating to the heart (e.g., fast, pounding or irregular heartbeat)

11. Headache

12. General weakness

13. Loss of taste or smell

14. Other
